# Extrauterine adenomyoma of the liver with a focally cellular smooth muscle component occurring in a patient with a history of myomectomy: case report and review of the literature

**DOI:** 10.1186/1746-1596-8-131

**Published:** 2013-08-05

**Authors:** Wu Huanwen, Zhang Hui, Xue Xiaowei, Lu Zhaohui

**Affiliations:** 1 Department of Pathology, Peking Union Medical College Hospital, Chinese Academy of Medical Science, 1 Shuaifuyuan, Dong Cheng District, Beijing 100730, China

**Keywords:** Extrauterine adenomyoma, Liver, Differential diagnosis, Pathogenesis

## Abstract

**Virtual Slides:**

The virtual slide(s) for this article can be found here: http://www.diagnosticpathology.diagnomx.eu/vs/1327125766102291.

Since first reported in 1986, 14 cases of extrauterine adenomyoma have been reported in the English literature, most often occurring in the ovaries. In this report, we present the first case of extrauterine adenomyoma involving the liver in a 29-year-old woman who presented with a 2-year history of low back pain with recent worsening and a history of laparoscopic myomectomy 5 years previously. Gross inspection of the specimen revealed a subcapsular mass that had a well-circumscribed margin with the adjacent liver tissue. By histopathologic examination, the multilobular mass was composed of a smooth muscle component and benign endometrioid glands and stroma. The smooth muscle component was focally cellular, and the endometrioid glands had secretory features. Both the smooth muscle component and endometrioid tissue were positive for ER and PR. The smooth muscle component was also positive for desmin and SMA, while the endometrioid stroma was positive for CD10. Other extrauterine lesions composed of a mixture of smooth muscle tissue and heterotopic endometrioid tissue, including endometriosis with a smooth muscle component, leiomyomatosis/leiomyomas associated with endometriosis and uterus-like masses, should be included in differential diagnoses. The patient was free from recurrence 5 months after liver tumor resection.

## Background

Adenomyomas are benign tumor-like masses composed of smooth muscle tissue and benign endometrioid glands and stroma. These tumors most commonly originate from within the uterine corpus. Adenomyomas in extrauterine sites are extremely rare. To the best of our knowledge, since it was first reported in 1986, only 14 cases of extrauterine adenomyoma have been reported in the English literature, most often occurring in the ovary [1-12].

In this report we describe the first case of extrauterine adenomyoma of the liver in a 29-year-old woman with a history of laparoscopic myomectomy. This is also the first case of a solitary extrauterine adenomyoma arising in an extra-pelvic site. We also present four major theories from the literature to explain the pathogenesis of extrauterine adenomyoma: Müllerian duct fusion defect, sub-coelomic mesenchyme transformation, müllerianosis and endometriosis with prominent smooth muscle hyperplasia or metaplasia.

## Case report

### Clinical findings

A 29-year-old married non-pregnant woman (P0G0), presented with a 2-year history of low back pain that had worsened over the past 2 months. There were no remarkable findings on physical examination. The patient had a history of uterine leiomyoma and had undergone laparoscopic myomectomy 5 years previously.

**Figure 1:**
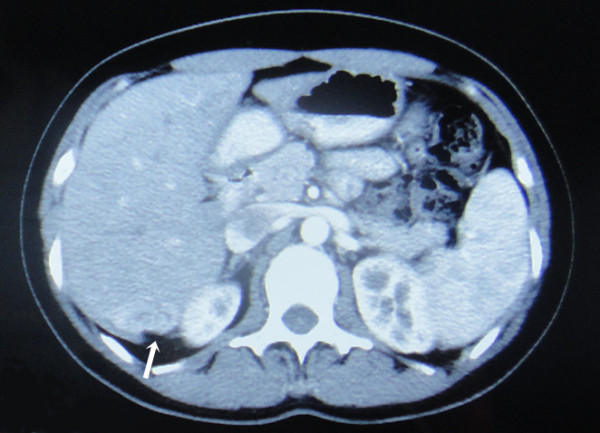
Contrast-enhanced CT showed a heterogeneously enhanced mass (arrow) in the peripheral zone of the posterior right lobe of the liver.

Abdominal ultrasonography revealed a 3.6 × 2.5 cm hypoechoic solid mass arising from the posterior right lobe of the liver. No other abnormality was detected on pelvic or abdominal ultrasonography. A subsequent CT scan demonstrated a patchy area with slightly lower density in the peripheral zone of the posterior right lobe of the liver. Contrast-enhanced CT showed heterogeneous enhancement of the lesion in the arterial phase (Figure  1). Laboratory investigations, including liver function tests and tumor markers (AFP, CEA, CA19-9, and CA125), were within normal ranges. Serological tests for hepatitis B surface antigen and anti-hepatitis C virus antibodies were negative.

Exploratory laparotomy revealed a solid, firm mass that was located at the subcapsular region in segment VI of the right liver near the right kidney. The rest of the liver and the other pelvic and abdominal organs appeared normal. The mass was completely removed by liver tumor resection. A frozen section was performed and interpreted as a spindle cell tumor, but primary and metastatic sarcoma of the liver could not be excluded. The patient was doing well and was free from recurrence 5 months after surgery.

### Pathology findings

**Figure 2:**
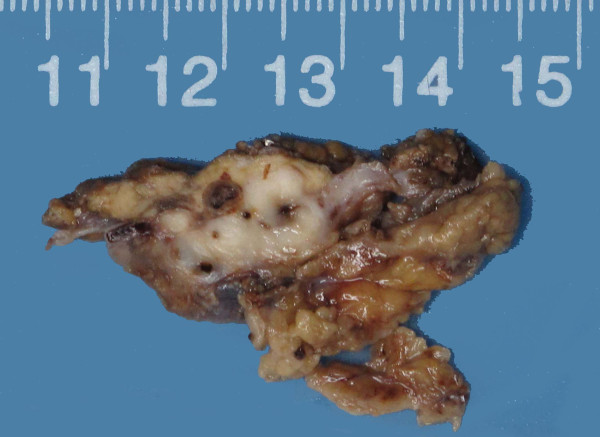
The cut surface showed a white-gray mass with cysts and foci of congestion and hemorrhage varying in diameter from 0.5 to 4 mm.

Gross inspection of the specimen revealed a 3.6 × 2.6x1.8 cm white-gray, irregular mass, which was partially covered with the hepatic capsule and partially surrounded by normal yellow-gray liver tissue. The mass was firm and solid, with scattered cysts and foci of congestion and hemorrhage up to 4 mm in diameter (Figure 2).

**Figure 3:**
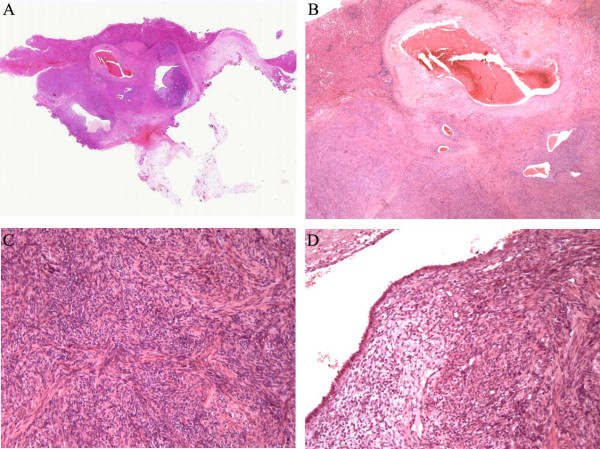
**Microscopic features (hematoxylin-eosin stain). A**, Histopathologic examination revealed a subcapsular mass. The multilobular mass had a well-circumscribed margin with the adjacent liver tissue. **B**, Irregular endometrioid glands and cysts were haphazardly scattered among the smooth muscle bundles. Variable amounts of blood/congestion and hemorrhage, dense fibrosis, and hemosiderin-laden macrophages were associated with the endometrioid tissue. **C**, The whorled intersecting smooth muscle component was focally cellular. **D**, The endometrioid cysts were typically lined by a single layer of columnar cells and had secretory features.

Histopathologic examination revealed a subcapsular mass, which had a well-circumscribed margin with the adjacent liver tissue. The mass was multilobular and composed of smooth muscle and benign glands and stroma. The smooth muscle component consisted of whorled intersecting bundles of typical smooth muscle cells with bland nuclei and was focally cellular. However, significant atypia, mitotic activity or necrosis was not observed. Irregular glands and cysts were haphazardly scattered among the smooth muscle bundles. The glands and cysts varied in size and shape and were typically lined by a single or pseudostratified layer of columnar cells, similar to the normal endometrial glands. The endometrioid glands had secretory features and were surrounded by a rim of endometrioid stroma. Variable amounts of blood/congestion and hemorrhage, dense fibrosis and hyalinization, and hemosiderin-laden macrophages were associated with the endometrioid tissue (Figure  3). The adjacent liver tissue was almost normal.

**Figure 4:**
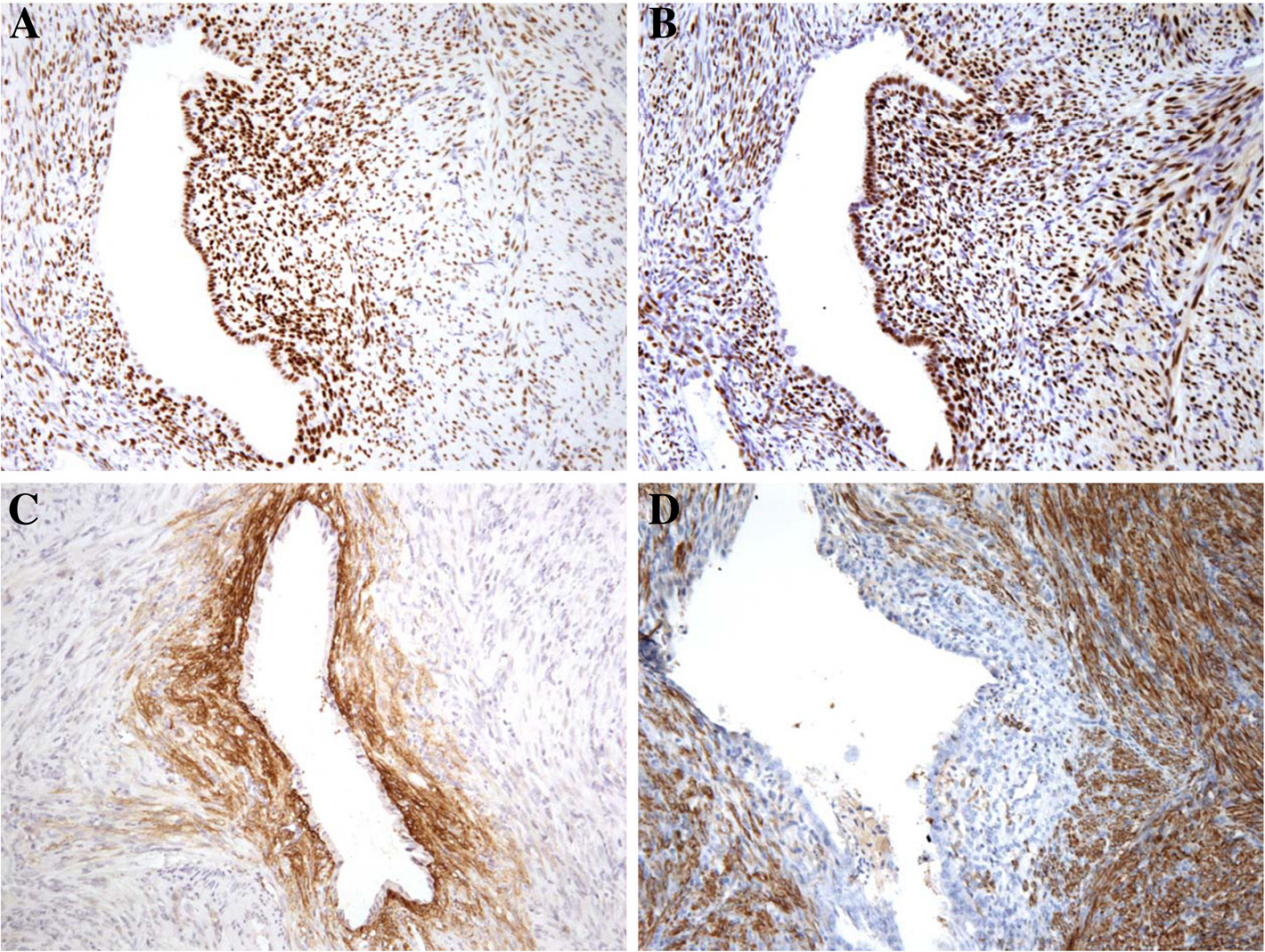
**Immunohistochemical staining was performed.** The smooth muscle component and the endometrioid tissue were both positive for ER **(A)** and PR **(B)**. The endometrioid stroma surrounding the endometrioid glands was positive for CD10 **(C)**, while the smooth muscle component was positive for desmin **(D)**.

Immunohistochemistry for CK7, ER, PR, SMA, desmin, CD10, S-100, CD34, CD117 and HMB45 was performed (Figure  4). The endometrioid glands were positive for CK7, ER and PR, while the endometrioid stroma surrounding the glands was positive for CD10, ER and PR and negative for SMA and desmin. The smooth muscle component was positive for ER, PR, SMA and desmin and negative for CD10, S-100, CD34, CD117 and HMB45.

Moreover, a review of slides from the previous laparoscopic myomectomy specimen showed a typical leiomyoma but no other significant pathology.

## Discussion

Endometriosis, which is defined as the presence of extrauterine endometrioid glands and stroma, is a common condition, with a prevalence of 5-10% in the reproductive female population. Endometriosis typically arises within the pelvis, including the fallopian tubes, ovaries and pelvic peritoneum. However, unusual extra-pelvic sites of endometriosis have also been reported, including the intestine, appendix, abdominal wall, skin, lung, bladder, umbilicus, kidney and even the central nervous system [13,14]. In an extensive review of the English literature, 20 cases of hepatic endometriosis have been described in premenopausal and postmenopausal women aged from 21-62 years old [15-22]. They most often presented with RUQ or epigastric pain, and approximately half had a prior history of pelvic endometriosis. The most common gross presentation of hepatic endometriosis is an endometrioid cyst called an “endometrioma”. The pathogenesis of hepatic endometriosis is still controversial, and blood/lymphatic dissemination is the presumed pathway for intraparenchymal hepatic lesions [15,17].

Extrauterine adenomyomas are defined as circumscribed tumor-like masses consisting of smooth muscle tissue and endometrioid glands and stroma and are similar in most respects to their more common uterine counterparts. They are much less common than endometriosis. To the best of our knowledge, only 14 cases of extrauterine adenomyoma have been reported in the English literature. Of these, 12 solitary cases were located within the pelvis, with 5 cases arising in the ovaries and 2 cases arising in the ovarian ligament. The other 2 cases involved multiple sites, including the ovaries, pelvic wall, mesentery, omentum, ileum and sigmoid colon [6]. In this report, we present the fifteenth case of extrauterine adenomyoma and the first case of hepatic adenomyoma. The 15 patients ranged from 29-65 (median 45.5) years of age, and the lesions had diameters of 0.4 cm to 10 cm. Most cases presented with low abdominal and pelvic pain, and 6 cases had a history of adenomyosis or pelvic endometriosis. The gross tumor appearance varied from solid and cystic to entirely solid. Unlike endometriosis, extrauterine adenomyoma is characteristically composed of both smooth muscle and endometrioid tissue. Microscopically, the endometrioid tissue is intermingled with bundles of smooth muscles. Both the smooth muscle component and the endometrioid tissue were benign in all 15 cases. However, multifocal nuclear atypia were reported in the smooth muscle component in 2 cases of extrauterine adenomyoma [5], and a focally cellular but benign smooth muscle component was described in our case.

Extrauterine adenomyomas could easily be misinterpreted as endometriosis or spindle cell tumors, including GISTs and leiomyomas/leiomyosarcomas, in intraoperative frozen sections because of insufficient sampling. In the formalin-fixed, paraffin-embedded sections of the resected tumor specimen, extrauterine adenomyomas should be first differentiated from other extrauterine lesions that are composed of a mixture of smooth muscle tissue and heterotopic endometrioid tissue, including endometriosis with a smooth muscle component, leiomyomatosis peritonealis disseminata/leiomyomas associated with endometriosis, and uterus-like masses. Endometriosis with a smooth muscle component might represent the hyperplasia/hypertrophy of indigenous smooth muscle or smooth muscle metaplasia within endometriosis [5]. Unlike endometriosis with indigenous smooth muscle, the lesion in our case had no obvious continuity with normal smooth muscle tissue, such as the fallopian tube or bowel wall, and was arranged in disorganized short fascicles and bundles. The smooth muscle metaplasia within endometriosis is typically focal and minor, but extrauterine adenomyomas show a dominant smooth muscle component and are more circumscribed than endometriosis both grossly and microscopically. Leiomyomatosis/leiomyomas have been reported to be associated with endometriosis. In most cases of leiomyomatosis/leiomyomas with endometriosis, the endometriotic cyst is either separated from the smooth muscle component or focally and peripherally admixed in the smooth muscle component. Moreover, the smooth muscle component might form multiple nodules [23-27]. In contrast, endometrioid glands and cysts are scattered within the smooth muscle tissue in adenomyoma, which typically forms a solitary nodule. Uterus-like masses are defined as extrauterine organoid masses in various sites, including the broad ligament, ovary and small intestine, that are characterized by a single central cavity lined by endometrium and surrounded by a thick wall of smooth muscle, resembling a normal uterus and most likely representing a particular form of extrauterine adenomyoma [6,28-32]. However, adenomyomas are typically with dispersed endometrioid glands and cysts and without an organoid arrangement with a single central cavity. It should be emphasized that the histological findings of these lesions are strikingly similar and may have overlapping features. Both adenomyomas with uterus-like features and uterus-like masses with adenomyoma features have been reported [2,9]. Leiomyomatosis with uterus-like features and multiple extrauterine adenomyomas/uterus-like masses resembling leiomyomatosis have also been described [6,23]. Therefore, these lesions may belong to a related entity of the same origin. Moreover, the histological distinction between theses lesions is not always clear, and the classification of such lesions may be somewhat arbitrary.

Extrauterine adenomyoma should also be differentiated from other tumors with combinations of smooth muscle tissue and epithelial components. Hepatobiliary adenomyoma is defined as a rare benign tumor-like lesion of the extrahepatic bile ducts, most often involving the gallbladder. A few cases have also been reported elsewhere in the gastrointestinal tract, bile ducts and ampullary region [33,34]. Microscopically, small glands lined by columnar cells are scattered among the fibromuscular stroma. The epithelial component in the hepatobiliary adenomyoma is similar to that of the normal biliary and pancreatic duct system. In contrast, our case demonstrated no relationship with extrahepatic bile ducts, and the epithelial component in our case was endometrioid glands with endometrioid stroma. Leiomyoma, GIST and PEComas with entrapped portal tracts might also be excluded. In these lesions, intrahepatic bile ducts should be found at the tumor periphery, and no endometrioid tissue should be detected. Moreover, GISTs and PEComas typically demonstrate positive immunoreactivity for CD117 and HMB45, respectively.

Another differential diagnosis was extrauterine benign metastasizing leiomyoma (BML) according to the immunohistochemical staining (ER/PR positive) and the history of myomectomy. However, BML is most frequently involving the lung [35], and endometrioid tissue is not observed in BML.

The morphologic criteria for distinguishing extrauterine benign Müllerian-type smooth muscle component from leiomyosarcoma are not well described. The current approach is to apply criteria of the corpus uteri to their extrauterine counterparts, and several potential indicators have been reported to be helpful in the differentiation [36-39].

The pathogenesis of extrauterine adenomyomas or uterus-like masses remains uncertain. We present four major theories from the literature. he Müllerian duct fusion defect theory has been proposed to explain the etiology [2,4-6]. This theory was supported by cases that have been associated with congenital urogenital abnormalities, including renal agenesis, a double excretory system and anomalies of the lower genital tract. The sub-coelomic mesenchyme transformation theory has become more popular in recent years [2,6,7,11,30]. The sub-coelomic mesenchyme or secondary Müllerian system is defined as the layer of tissue that lies underneath the mesothelial surface of the pelvic and lower abdominal peritoneum. According to this theory, extrauterine adenomyomas/uterus-like masses are derived from the sub-coelomic mesenchyme or so-called “secondary Müllerian system”, which is supported by cases without congenital anomalies and the effectiveness of hormone-related therapy in controlling the disease. In further support of this theory, most cases of extrauterine adenomyomas/uterus-like masses arise from pelvic and lower abdominal structures. The müllerianosis (developmentally misplaced Müllerian tissue) theory has provided another explanation for the pathogenesis of extrauterine lesions composed of both smooth muscle and endometrioid tissue. Müllerianosis was defined by Batt as a heterotopic organoid structure composed of Müllerian rests (endometrial tissue, endosalpingeal tissue and/or endocervical tissue) that were incorporated within other normal organs during organogenesis [40,41]. The müllerianosis theory was particularly suitable for providing an explanation for extrauterine lesions that occurred in unusual sites outside the pelvic and lower abdominal cavities, such as the spinal cord [42]. This theory was further supported by the presence of ectopic endometrium in human fetuses and lesions that presented in patients without evidence of pelvic endometriosis or a history of surgery on the reproductive organs [40,41]. Endometriosis with prominent smooth muscle hyperplasia or metaplasia, which has also been called endomyometriosis, is the fourth theory to explain pathogenesis [7,43]. Due to the different opinions on their origin and pathogenesis, different diagnoses, including extrauterine adenocarcinoma/uterus-like mass, müllerianosis and endomyometriosis, might be reached for the same case by different pathologists [41,42,44]. The first three theories all agree that these lesions are directly derived from multi-potential cells of either the Müllerian or second Müllerian system. The inducing factors of benign Müllerian lesions arising from multi-potential cells remain to be determined. Hormonal stimulation was considered as one of the major inducing factors [2,6]. Because approximately half of the cases of extrauterine adenomyomas, including our case, have a history of surgery on the reproductive organs 5-22 years previously, surgical stimulation might also be one of the inducing factors [2,5,6,9]. It is more likely that our case arose from the tissue of the secondary Müllerian system because no evidence of adenomyosis, pelvic endometriosis, endosalpingiosis, endocervicosis or congenital urogenital abnormalities was found, and the lesion was located at the liver subcapsular region, which was partially covered by the hepatic peritoneum.

The preoperative diagnosis of extrauterine adenomyoma is difficult because of its rarity and non-specific clinical and radiological findings. Primary or metastatic malignancies should be included in the preoperative differential diagnosis. Moreover, although the malignant transformation of extrauterine adenocarcinomas has not been reported, it is an uncommon but possible event in endometriosis, particularly in liver endometrioma. For example, one case of in situ adenocarcinoma, one case of adenosquamous carcinoma and two cases of adenosarcoma were considered to arise in the setting of liver endometrioma [19-22]. A uterus-like mass associated with endometrioid carcinoma has also been described. Therefore, complete tumor resection might be recommended for extrauterine adenocarcinoma. All 15 reported cases underwent complete tumor resection. Only one case with multiple lesions relapsed, which occurred 1 year after tumor resection; the patient received monthly GnRH agonist treatment, and the disease was well controlled during 10 years of follow-up [6].

## Conclusion

In summary, adenomyomas in extrauterine sites are extremely rare. We present the first case of an extrauterine adenocarcinoma with a cellular smooth muscle component involving the liver. Such masses could be easily misinterpreted in the preoperative diagnosis and intraoperative frozen diagnosis. The resected tumor specimen should first be differentiated from other extrauterine lesions, which are composed of a mixture of smooth muscle tissue and heterotopic endometrioid tissue. In this case, the sub-coelomic mesenchyme theory was favored for pathogenesis, and we propose that differentiation from sub-coelomic mesenchyme to adenocarcinoma may be induced by hormonal and/or surgical stimulation.

## Consent

To publish this case report and accompanying images, written informed consent was obtained from the patient’s family.

## Competing interests

The authors declare that they have no competing interests.

## Authors’ contributions

HW W was the main author on the paper, took the clinical images, worked up the case and drafted the manuscript. H Z and XW X conducted the immunohistochemical study. ZH L was the main pathologist involved in the case, made the finally diagnosis and was the main editor of the body of the text. All authors read and approved the final manuscript.
